# Differential regulation of platelet inhibition by cGMP- and cAMP-dependent protein kinases

**DOI:** 10.1186/2050-6511-14-S1-P69

**Published:** 2013-08-29

**Authors:** Hariharan Subramanian, Ulrich Walter, Stepan Gambaryan

**Affiliations:** 1 Institute of Clinical Biochemistry& Pathobiochemistry, University of Wuerzburg, Wuerzburg, Germany; 2 Center for Thrombosis and Hemostasis (CTH), Johannes Gutenberg University, Mainz, Germany; 3 Sechenov Institute of Evolutionary Physiology and Biochemistry, Russian Academy of Sciences, St. Petersburg, Russia

## Background

Under normal conditions circulating platelets are maintained in a resting state by the paracrine signaling molecules, prostacyclin (PGI_2_) and nitric oxide (NO), released from vascular endothelial cells. PGI_2_ and NO trigger the synthesis of cAMP and cGMP, respectively, which in turn leads to the activation of Protein Kinase A (PKA) and Protein Kinase G (PKG). These cyclic nucleotide-dependent protein kinases phosphorylate numerous substrate proteins involved in several aspects of platelet activation. Both, PKA and PKG can phosphorylate the same substrates including VASP, LASP, RAP1GAP2, Rap1B, HSP27, and some others. Only few substrates are known to be specific for PKA (PDE3) and PKG (PDE5) [1,2]. Here we report the identification and characterization of a new PKA specific substrate, CalDAG-GEFI (calcium and diacylglycerol regulated guanine nucleotide exchange factor I), a guanine exchange factor for Rap1b in platelets.

## Results

Using radioactive phosphate incorporation assay, we identified S587 as the major PKA phosphorylation site in CalDAG-GEFI, which was confirmed by mass spectrometry. In platelets, PKA stimulation leads to strong phosphorylation of CalDAG-GEFI and inhibition of Rap1b activation triggered by thrombin, as well as calcium ionophore, which indicates a potential role of CalDAG-GEFI phosphorylation in the inhibition of Rap1b activation. However, PKG activation did not significantly phosphorylate CalDAG-GEFI, and inhibits thrombin-stimulated Rap1b by inhibition of calcium mobilization but did not inhibit calcium ionophore-triggered Rap1b activation. In HEK293 cells transfected with CalDAG-GEFI, calcium ionophore triggered Rap1b activation and forskolin stimulated PKA phosphorylation of CalDAG-GEFI leads to inhibition of Rap1b activation. But in cells with S587A mutant, PKA activation did not inhibit Rap1b activation. The phospho-mimetic (S587D) mutant failed to activate Rap1b following calcium ionophore stimulation.

## Conclusion

These results confirmed that PKA phosphorylation of CalDAG-GEFI inhibits Rap1b activation. We propose that this is one of the important mechanisms of platelet inhibition mediated by cAMP/PKA signaling. Presented data show that PKA and PKG could utilize similar and/or diverse intracellular mechanisms involved in platelet inhibitory pathways.
